# Influence of circulating nesfatin-1, GSH and SOD on insulin secretion in the development of T2DM

**DOI:** 10.3389/fpubh.2022.882686

**Published:** 2022-08-15

**Authors:** Kangkang Huang, Yunlai Liang, Kun Wang, Jiahui Wu, Huidan Luo, Bin Yi

**Affiliations:** ^1^Department of Clinical Laboratory, Xiangya Hospital, Central South University, Changsha, China; ^2^National Clinical Research Center for Geriatric Disorders, Xiangya Hospital, Central South University, Changsha, China

**Keywords:** type 2 diabetes mellitus, prediabetes, GSH, SOD, insulin secretion

## Abstract

**Aims:**

To evaluate the correlation of nesfatin-1, GSH and SOD levels with β-cell insulin secretion and their influence on insulin secretion in the development of type 2 diabetes mellitus (T2DM).

**Materials and methods:**

75 patients with T2DM, 67 with prediabetes and 37 heathy participants were recruited in this study. Serum levels of nesfatin-1, GSH and SOD were quantified and statistically analyzed.

**Results:**

The levels of nesfatin-1, GSH and SOD in T2DM were significantly decreased (*P* < 0.001) compared to either in prediabetes or in healthy control, and significant reduction of these biomarkers was also observed in prediabetes when compared to the control (*P* < 0.001). Circulating nesfatin-1, GSH and SOD were not only strongly correlated with β-cell insulin secretion, but also exerted remarkable influence on the secretion.

**Conclusion:**

Serum nesfatin-1, GSH and SOD are important factors involving insulin secretion in the development of T2DM, which may help provide new ideas for forthcoming investigations on the roles of these factors in pathogenesis of T2DM, as well as for active prediction and prevention of prediabetes before it develops into overt T2DM.

## Introduction

Up to 2021, the global prevalence of diabetes reached 10.5% (536.6 million people) and the number is estimated up to 12.2% (783.2 million people) by 2045 (1); among them, 90% to 95% are type 2 diabetes mellitus (T2DM) (2). Prediabetes is a risky stage before T2DM, characterized by metabolic abnormality of the body, such as impaired fasting glucose (IFG) or impaired glucose tolerance (IGT). A meta-analysis concluded that the hazard ratios for IFG, IGT and IFG+IGT developing into T2DM are 4.32, 3.61 and 6.90, respectively (3).

Although resistance of peripheral tissues to insulin or β-cell dysfunction is common in T2DM, the exact mechanism of T2DM remains to be clarified. Multiple explanations have been proposed in the development of T2DM, of them, oxidative stress is considered to be pivotal in this process (4). Free radicals, including reactive oxygen species (ROS), and some metal ions (such as iron and copper) can be generated through metabolic pathways or immune cells (5, 6), and play key roles in many physiological activities such as cell signaling, growth, apoptosis and aging (7–9). When free radicals are accumulated, they will overcome the anti-oxidative effects in the cell, initiated by such as glutathione (GSH) or superoxide dismutase (SOD), resulting in oxidative stress (9, 10). Pancreatic β-cells heavily rely on oxidative metabolism to synthesize adenosine triphosphate, especially when the glucose level is high (11, 12). In spite of the fact that pancreatic β-cells actively function in metabolic process, which leads to ROS accumulation as ROS is an inevitable byproduct of mitochondrial respiration during glucose stimulation (13), enzymes involved in anti-oxidative defenses are present at very low levels in β-cells and they are prone to be inactivated by disallowed genes (11); in this regard, protecting pancreatic β-cells from the destructive free radicals is expected to be a potential strategy for preventing and controlling T2DM.

Besides insulin, many different peptide hormones such as glucagon-like peptide-1 (GLP-1) and glucose-dependent insulinotropic polypeptide (GIP) can affect the balance of glucose metabolism in the body (14). GLP-1 and GIP are released into the circulation from gut endocrine cells in response to food digestion and facilitate insulin secretion in a glucose-dependent manner (15, 16). However, the very short half-lives (1–7 min) of GLP-1 and GIP in plasma represent a major limitation for their use in the clinical setting (17). Nesfatin-1 is a newly identified peptide with 82 amino acids; in addition to nesfatin-1, cleavage of prohormone convertase on NEFA/nucleobindin2 (NUCB2) yields fragments of nesfatin-2 and nesfatin-3 (18). Although its receptor is still unclear, nesfatin-1 has been found to be functional in anti-inflammation (19), antioxidation (20), appetite suppression (21) and insulin resistance (22). Importantly, researches on the variation of serum nesfatin-1 levels in T2DM have so far proved inconclusive. Some studies reported elevated serum nesfatin-1 levels (23, 24), while others showed the contrary results (25–27). Another meta-analysis concluded that serum nesfatin-1 upregulated in newly diagnosed T2DM patients but decreased after drug therapy (28).

Homeostatic model assessment (HOMA) is a convenient and economic method to quantify β-cell function of insulin secretion (HOMA-β), insulin resistance (HOMA-IR) and insulin sensitivity (HOMA-IS) with measurement of fasting blood glucose and insulin (29).

We conducted this cross-sectional study to assess the correlation of nesfatin-1, GSH and SOD levels with β-cell insulin secretion, and to explore their influence on insulin secretion in the development of T2DM through prediabetes.

## Materials and methods

### Participants

**Figure 1:**
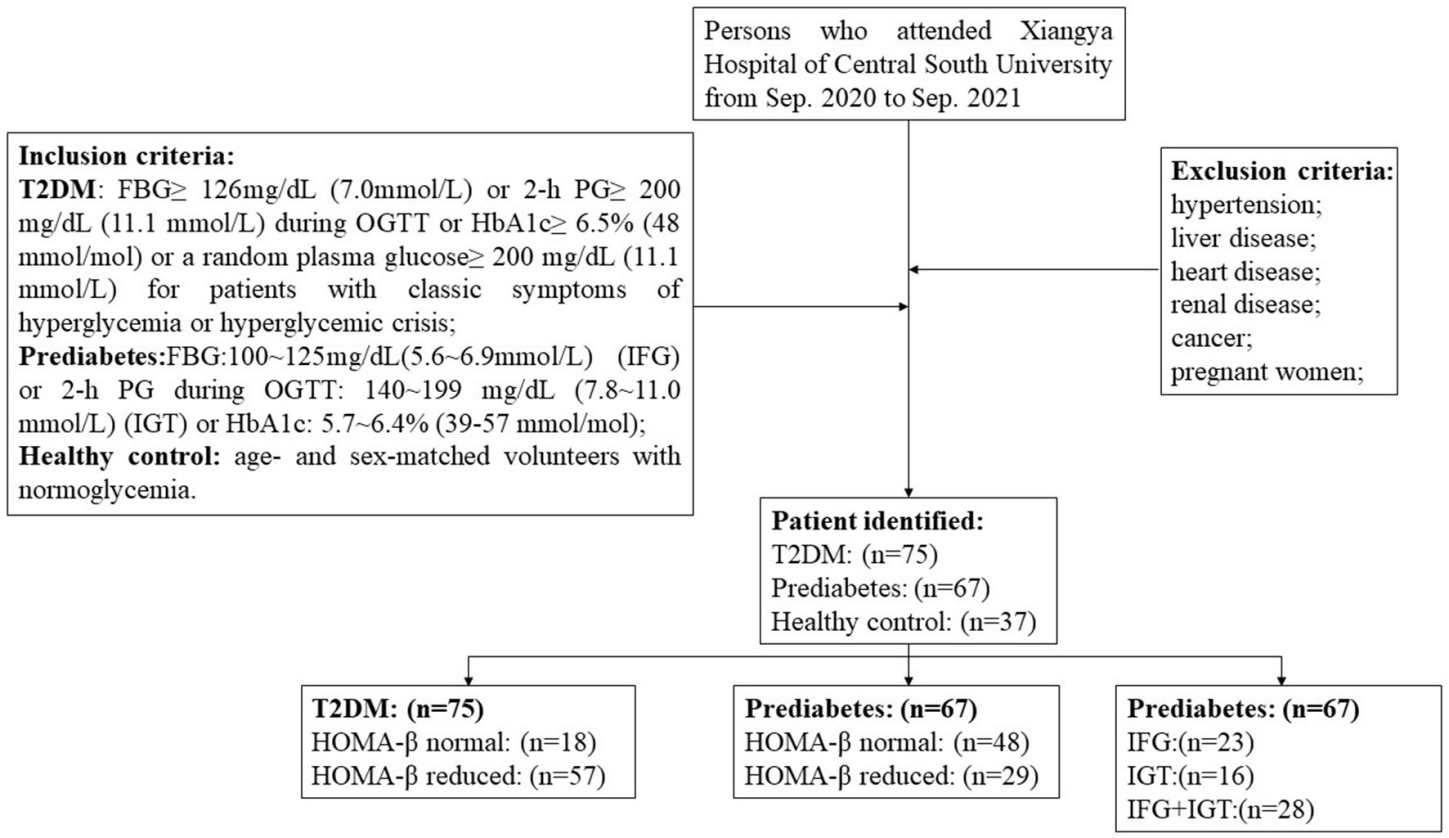
Flow chart depicting patient selection.

This cross-sectional study recruited 75 T2DM patients, 67 prediabetes who attended in Xiangya Hospital of Central South University from Sep. 2020 to Sep. 2021. According to the American Diabetes Association (ADA) guideline for diabetes (30), the inclusion criteria for T2DM include the following: FBG ≥ 126 mg/dL (7.0 mmol/L) or 2-h PG ≥ 200 mg/dL (11.1 mmol/L) during oral glucose tolerance test (OGTT) or HbA1c ≥ 6.5% (48 mmol/mol) or a random plasma glucose ≥ 200 mg/dL (11.1 mmol/L) for patients with classic symptoms of hyperglycemia or hyperglycemic crisis; the criteria required for prediabetes inclusion contain FBG: 100~125 mg/dL (5.6~6.9 mmol/L), IFG or 2-h PG during 75-g OGTT: 140~199 mg/dL (7.8~11.0 mmol/L) (IGT) or HbA1c: 5.7~6.4% (39–57 mmol/mol). 37 age- and sex-matched volunteers with normoglycemia were introduced as the healthy controls. Subjects with hypertension, liver disease, heart disease, renal disease, cancer, or other chronic diseases as well as pregnant women were excluded. The procedure of patient selection was depicted in a flowchart (Figure 1). All participants were given informed consent and this study was permitted by the ethics committee of Xiangya Hospital of Central South University (No. 202109180).

Blood samples were drawn between 08:00 a.m. and 10:00 a.m. from each participant after fasting food for at least 8 h. Body weight and height were assessed and body mass index (BMI) was calculated by dividing weight in kilograms by height in meters squared. The collected venous blood samples were centrifuged at 3,600 rpm for 10 min to isolate sera and stored at −20 °C until they were required for testing. Routine tests for biochemistry indicators such as high-density lipoprotein cholesterol (HDL-C), low-density lipoprotein cholesterol (LDL-C), total bile acid (TBA), uric acid (UA), serum creatinine (Scr), blood urea nitrogen (BUN), glycosylated hemoglobin (HbA1c), fasting blood-glucose (FBG), insulin, urine creatinine (Ucr) and urine microalbumin (UmALB) were measured on an AU5800 automatic analyzer (Beckman Coulter, CA, USA). HOMA-β, HOMA-IR and HOMA-IS were calculated by the following equations (29): HOMA-β = 20 ^*^ insulin (μU/ml)/(FBG(mmol/L)-3.5); HOMA-IR = insulin (μU/ml) ^*^ FBG(mmol/L)/22.5 and HOMA-IS = 100 ^*^ 22.5/insulin (μU/ml) ^*^ FBG(mmol/L).

Serum levels of nesfatin-1, GSH and SOD were determined using commercially available kits in accordance with the manufacturers' instructions. Nesfatin-1 was measured by a double-antibody sandwich enzyme-linked immunosorbent assay (ELISA), supplied by Jiangsu Meimian Industrial, Jiangsu, China. In brief, 50 μL of the 1:5 diluted serum specimens were added to each microplate well pre-coated with purified human nesfatin-1 antibody, and incubated at 37°C for 30 min; after washed 5 times with washing buffer, 50 μL of HRP-conjugated nesfatin-1 antibody was added and kept at 37°C for another 30 min; following 5 repeatedly washing steps, 50 μL of the TMB substrate solution A and 50 μL of the substrate B were pipetted to each well and preserved at 37°C from light for 10 min; finally, 50 μL of stop solution were added to terminate the reaction. The absorbance at 450 nm (A_450_) of each well was read within 15 min on an automatic microplate reader and the concentration of nesfatin-1 is quantified by comparing the A_450_ of the samples to the standard curve.

Detection of GSH is based on an enzymatic cycling method in the presence of GSH and a chromophore, and the assay kit was provided by Nanjing Jiancheng Bioengineering Institute, Nanjing, China. The reduction of the chromophore produces a stable product, which can be followed by measuring A_405_, therefore, the A_405_ is directly proportional to the amount of GSH in the sample. The procedure began with adding 50 μL of the serum into 200 μL of the precipitant working solution, followed by centrifugation at 3,500 rpm for 10 min before 100 μL of the supernatant were collected, then 100 μL of the GSH assay buffer and 25 μL of the chromogenic agent were added to the supernatant with sufficient mixing, kept at room temperature from light for 5 min, after that, the A_405_ of each well was read within 10 min on an automatic microplate reader and the levels of GSH was derived from the prepared standard curve.

The SOD WST-1 assay kit (Nanjing Jiancheng Bioengineering Institute, China) allows a very convenient and highly sensitive SOD measurement by utilizing WST-1 (2-(4-iodophenyl)-3-(4-nitrophenyl)-5-(2,4-disulfo-phenyl)-2H-tetrazolium, monosodium salt), which produces a water-soluble formazan dye upon reduction with a superoxide anion, and the reduction is linearly related to the xanthine oxidase activity and is inhibited by SOD. Therefore, the IC50 (50% inhibition concentration) of SOD can be determined using colorimetric methods. In brief, 20 μL of the serum sample and 20 μL of the enzyme working solution were pipetted to the sample well, followed by adding 200 μL of the WST working solution, then incubated at 37°C for 20 min. Meanwhile, blank 1 (coloring without inhibitor), blank 2 (sample blank) were prepared as indicated in the manufacturer's manual. The absorbance at 450 nm of each well was read within 10 min on a microplate reader, and the activity of SOD was calculated with the following equation: SOD activity (U ml^−1^) = (A _blank1_ – A _sample_)/(A _blank1_ – A _blank2_) × 40.

Appropriate kits for testing serum adiponectin (ADPN) (Guangdong Uniten Biotechnology, Guangdong, China), retinol binding protein (RBP) (Aucher, Hunan, China), total iron binding capacity (TIBC) (Beijing Strong Biotechnologies, Beijing, China), neutrophil gelatinase-associated lipocalin (NAGL) (Aucher, Hunan, China) and cystatin C (CysC) (Aucher, Hunan, China) were adopted for quantification of the above indicators.

### Statistical analysis

Statistical analysis was implemented using SPSS version 26 (SPSS Inc., IL, USA). The results for continuous variables were presented as mean ± standard deviation (SD) and underwent normal distribution test, while the parameter of age was shown as median. Differences among groups were calculated with ANOVA, meanwhile, differences between groups were evaluated with SNK test. Independent Samples *t*-Test was used to determine the differences between two unpaired subgroups. Gender as categorical data was coded as male = 1 and female = 0. Differences of gender and age were acquired by Chi-Square test. Correlations between HOMA-β and other indexes were analyzed with Pearson correlation test. The impact factors of HOMA-β were assessed with multiple linear regression analysis (α in = 0.05, α out = 0.10). *P* < 0.05 (two-tailed) was regarded as statistically significant.

## Results

### General characteristics and parameter comparisons

A total of 179 participants were recruited in this study, consisting of three groups: T2DM, prediabetes, and the healthy control. The anthropometric and clinical characteristics of the subjects were shown in Table 1 and there were no significant differences in gender, age and BMI among the three groups. After normality of the continuous variables was tested and validated, differences of the serum indicators were compared. The results showed that serum levels of nesfatin-1, GSH, SOD, ADPN and NAGL in T2DM were significantly decreased compared to either in prediabetes (*P* < 0.001) or in healthy controls (*P* < 0.001); in contrast, RBP levels in T2DM were significantly elevated (*P* < 0.001) compared to either in prediabetes or in healthy controls, and this significant elevation exhibited in the prediabetes vs. the healthy (*P* < 0.001). In addition, TIBC levels in T2DM were distinctly high (*P* < 0.01) in comparison with either in prediabetes or in healthy controls.

**Table 1:** Anthropometric and clinical characteristics of the study subjects in different groups.

**Parameters**	**T2DM**	**Prediabetes**	**Healthy control**
Gender (M/F)*	41/34	33/34	23/14
Age (years)^a^*	54 (51–61)	55 (52–59)	52 (47.5–57.5)
BMI (Kg/m^2^)^b^	23.01 ± 3.89	23.15 ± 2.76	22.94 ± 2.18
GSH (μmol/L)^b^	8.70 ± 3.60^###&&&^	12.04 ± 6.62^&&&^	14.97 ± 6.93
SOD (U/ml)^b^	1,577.12 ± 180.67^###&&&^	1,976.14 ± 234.00^&&&^	2,089.95 ± 190.66
Nesfatin-1 (pg/ml)^b^	622.94 ± 218.28^###&&&^	875.88 ± 578.71	1,060.43 ± 823.72
ADPN (μg/ml)^b^	17.76 ± 12.64^###&&&^	64.50 ± 12.84^&&&^	90.22 ± 7.21
RBP (mg/L)^b^	88.29 ± 31.83^###&&&^	32.89 ± 17.59^&&&^	14.39 ± 3.27
TIBC (μmol/L)^b^	67.30 ± 61.85^##&&^	39.72 ± 8.39	42.42 ± 28.79
NAGL (ng/ml)^b^	450.47 ± 232.53^###&&&^	1,344.44 ± 365.46^&&&^	2,512.84 ± 654.34
CysC (mg/L)^b^	0.88 ± 0.76	0.70 ± 0.16	0.67 ± 0.16
HDL-C (mmol/L)^b^	1.16 ± 0.29^#&^	1.32 ± 0.27	1.30 ± 0.24
LDL-C (mmol/L)^b^	3.30 ± 0.99	3.58 ± 0.74	3.35 ± 0.72
HDL-C/LDL-C^b^	0.39 ± 0.20	0.38 ± 0.11	0.41 ± 0.12
TBA (μmol/L)^b^	3.48 ± 3.25	3.53 ± 3.92	2.89 ± 2.35
UA (μmol/L)^b^	349.12 ± 114.25	364.57 ± 87.24	373.45 ± 82.19
Scr (μmol/L)^b^	1.37 ± 1.80	0.92 ± 0.17	0.94 ± 0.16
BUN (mmol/L)^b^	8.48 ± 16.94	5.31 ± 1.13	6.48 ± 8.89
HbA1c (%)^b^	7.83 ± 1.92^##&&^	5.88 ± 0.32	5.56 ± 0.32
FBG (mmol/L)^b^	8.36 ± 3.45^##&&^	5.86 ± 0.42	5.26 ± 0.53
2h-PG (mmol/L)^b^	13.62 ± 4.44^##&&^	8.66 ± 1.55^&&^	6.44 ± 0.90
Insulin (μIU/ml)^b^	6.87 ± 3.74^#^	9.50 ± 6.45^&^	6.46 ± 3.26
HOMA-IR^b^	2.41 ± 1.33^&^	2.48 ± 1.66^&^	1.55 ± 0.88
HOMA-β^b^	38.62 ± 28.49^###&&&^	83.29 ± 62.61	74.87 ± 34.00
HOMA-IS^b^	0.58 ± 0.45	0.55 ± 0.29	0.89 ± 0.58

^a^Median (interquartile range), ^b^mean ± SD, ^*^Chi-Square P < 0.05. BMI, body mass index; GSH, glutathione; SOD, superoxide dismutase; ADPN, adiponectin; RBP, retinol binding protein; TIBC, total iron binding capacity; NAGL, neutrophil gelatinase-associated lipocalin; CysC, cystatin C; HDL-C, high density lipoprotein cholesterol; LDL-C, low density lipoprotein cholesterol; TBA, total bile acid; UA, uric acid; Scr, serum creatinine; BUN, blood urea nitrogen; HbA1c, glycosylated hemoglobin; FBG, fasting blood-glucose; 2h-PG, two-hour post glucose; HOMA-IR, homeostasis model assessment of insulin resistance; HOMA-β, homeostasis model assessment of β-cell; HOMA-IS, homeostasis model assessment of insulin sensitivity. ^#^: vs. prediabetes P < 0.05, ^##^: vs. prediabetes P < 0.01, ^###^: vs. prediabetes P < 0.001; ^&^: vs. healthy control P < 0.05, ^&&^: vs. healthy control P < 0.01, ^&&&^: vs. healthy control P < 0.001.

### Correlation between HOMA-β and other indexes

**Figure 2:**
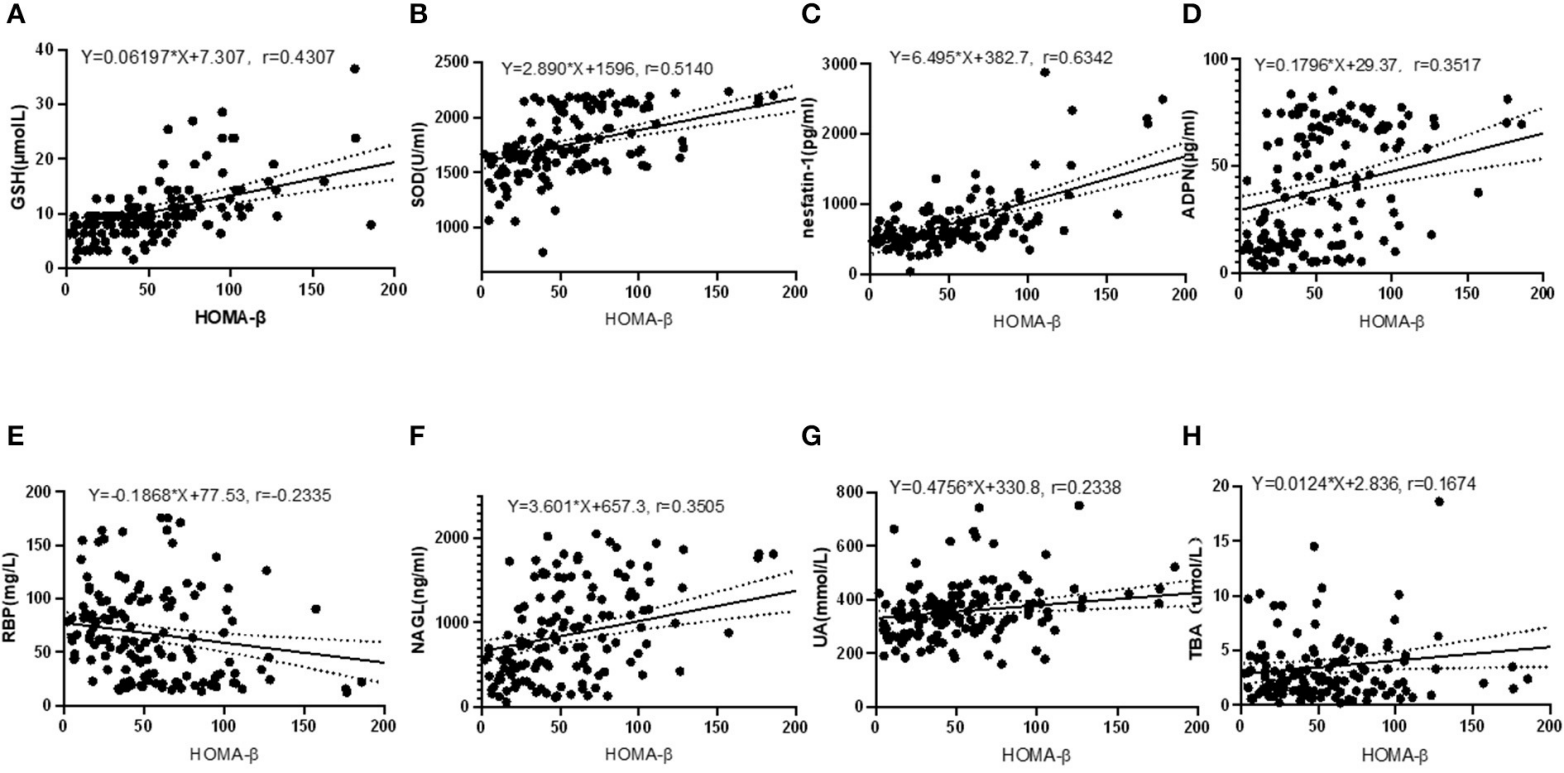
Correlations between HOMA-β and GSH, SOD, nesfatin-1, ADPN, RBP, NAGL, UA and TBA. **(A)** correlation between HOMA-β and GSH (*R*^2^ = 0.1855, *F* = 31.88, *P* < 0.001); **(B)** correlation between HOMA-β and SOD (*R*^2^ = 0.2642, *F* = 50.26, *P* < 0.001); **(C)** correlation between HOMA-β and nesfatin-1(*R*^2^ = 0.4022, *F* = 94.18, *P* < 0.001); **(D)** correlation between HOMA-β and ADPN (*R*^2^ = 0.1237, *F* = 19.77, *P* < 0.001); **(E)** correlation between HOMA-β and RBP (*R*^2^ = 0.0545, *F* = 8.070, *P* = 0.005); **(F)** correlation between HOMA-β and NAGL (*R*^2^ = 0.1299, *F* = 19.61, *P* < 0.001); **(G)** correlation between HOMA-β and UA (*R*^2^ = 0.0547, *F* = 8.097, *P* = 0.005); **(H)** correlation between HOMA-β and TBA (*R*^2^ = 0.0280, *F* = 4.034, *P* = 0.046).

**Figure 3:**
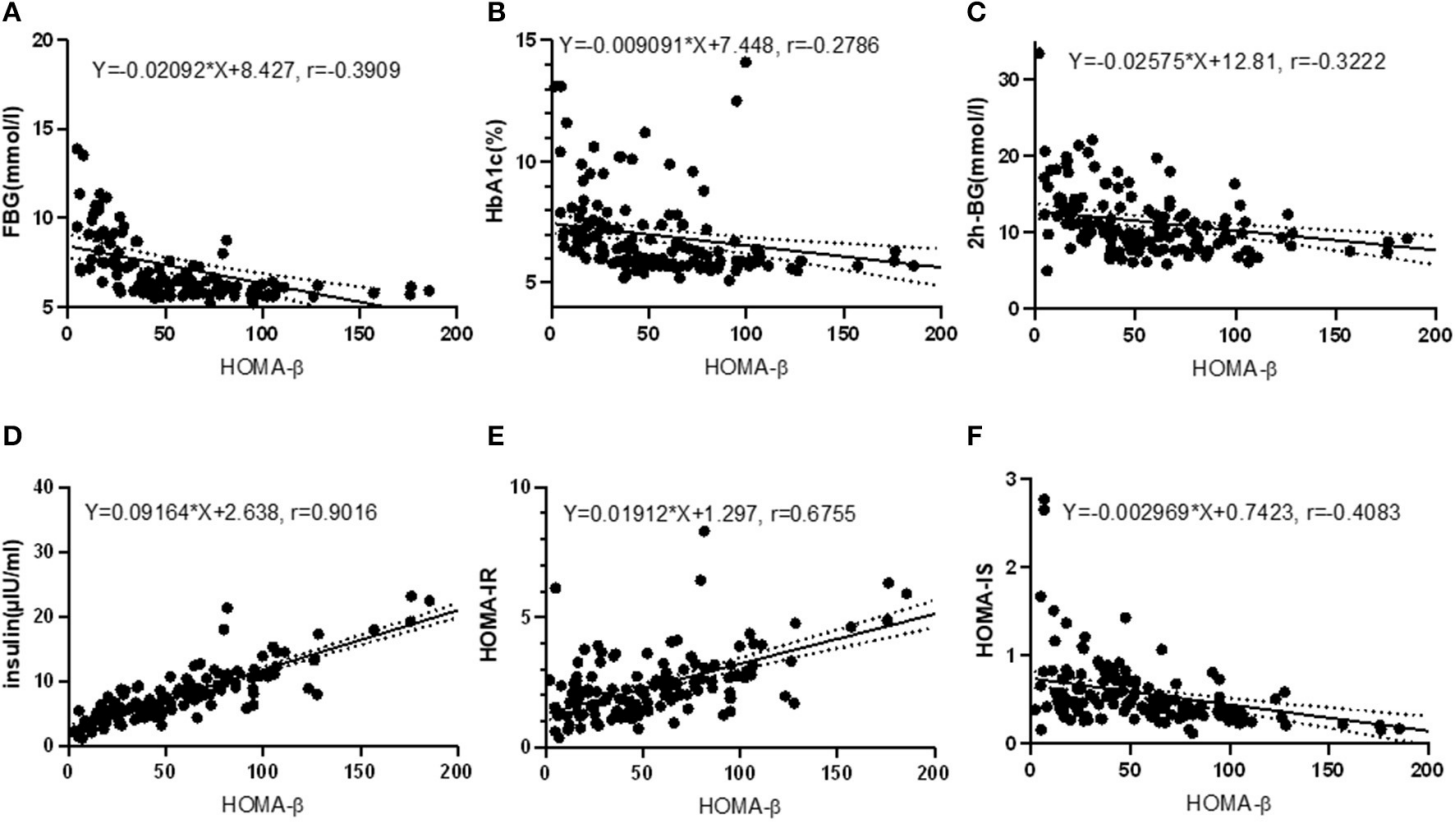
Correlations between HOMA-β and glucose homeostasis indexes. **(A)** correlation between HOMA-β and FBG (*R*^2^ = 0.1528, *F* = 25.25, *P* < 0.001); **(B)** correlation between HOMA-β and HbA1c (*R*^2^ = 0.0800, *F* = 11.78, *P* < 0.001); **(C)** correlation between HOMA-β and 2h-BG (*R*^2^ = 0.1038, *F* = 16.21, *P* < 0.001); **(D)** correlation between HOMA-β and insulin (*R*^2^ = 0.8128, *F* = 608.0, *P* < 0.001); **(E)** correlation between HOMA-β and HOMA-IR (*R*^2^ = 0.4563, *F* = 117.5, *P* < 0.001); **(F)** correlation between HOMA-β and HOMA-IS (*R*^2^ = 0.1667, *F* = 28.01, *P* < 0.001).

Pearson correlation test disclosed that HOMA-β was closely correlated with serum GSH (*r* = 0.4307, *P* < 0.001), SOD (*r* = 0.5140, *P* < 0.001), nesfatin-1 (*r* = 0.6342, *P* < 0.001), ADPN (*r* = 0.3517, *P* < 0.001), RBP (*r* = −0.2355, *P* = 0.005), NAGL (*r* = 0.3505, *P* < 0.001), UA (*r* = 0.2338, *P* = 0.005) and TBA (*r* = 0.1675, *P* = 0.046), as shown in Figure 2. Relevance analysis between HOMA-β and the glucose metabolism indexes (Figure 3) found that HOMA-β was significantly relevant to FBG (*r* = −0.3909, *P* < 0.001), HbA1c (*r* = −0.2786, *P* < 0.001), 2h-PG (*r* = −0.3222, *P* < 0.001), insulin (*r* = 0.9016, *P* < 0.001), HOMA-IR (*r* = 0.6755, *P* < 0.001) and HOMA-IS (*r* = −0.4083, *P* < 0.001).

### Impact factors on HOMA-β level

Multiple linear regression analysis was performed to evaluate the impact factors on β-cell insulin secretion, where HOMA-β was set as the dependent variable while the independent variables included GSH, SOD, nesfatin-1, ADPN, RBP, NAGL, UA, TBA, FBG, HbA1c, 2h-PG, insulin, HOMA-IR and HOMA-IS (α in = 0.05 and α out = 0.10 with backward selection). Factors of GSH, SOD, nesfatin-1, FBG, insulin, HOMA-IR and HOMA-IS were introduced to the equation as Y = 0.729X_1_+0.012X_2_+0.007X_3_+4.752X_4_+18.518X_5_-38.817X_6_+6.512X_7_-67.357, R^2^ = 0.951 (Y: HOMA-β, X1: GSH, X_2_: SOD, X_3_: nesfatin-1, X_4_: FBG, X_5_: insulin, X_6_: HOMA-IR, X_7_: HOMA-IS, *P* < 0.001), with the adjusted R^2^ = 0.948. The standardized regression coefficients of GSH, SOD, nesfatin-1, FBG, insulin, HOMA-IR and HOMA-IS were 0.076, 0.066, 0.056, 0.254,1.882, −1.099, 0.047, respectively (Table 2).

**Table 2:** Independent variables introduced to multiple liner regression.

**Parameters**	***b***	**S_b_**	**b'**	**|t|**	***P***
Constant	−67.36	9.36	/	7.195	0.000
GSH	0.73	0.21	0.076	3.413	0.001
SOD	0.012	0.004	0.066	2.941	0.004
Nesfatin-1	0.007	0.003	0.056	2.044	0.043
FBG	4.752	0.697	0.254	6.813	<0.001
Insulin	18.518	1.006	1.882	18.399	<0.001
HOMA-IR	−38.817	3.288	−1.099	11.805	<0.001
HOMA-IS	6.512	3.590	0.047	1.814	0.072

*GSH, glutathione; SOD, superoxide dismutase; FBG, fasting blood-glucose; HOMA-IR, homeostasis model assessment of insulin resistance; HOMA-IS, homeostasis model assessment of insulin sensitivity*.

Interestingly, when we excluded glucose metabolism indexes of FBG, HbA1c, 2h-PG, insulin, HOMA-IR and HOMA-IS, and included GSH, SOD, nesfatin-1, ADPN, RBP, NAGL, UA and TBA as independent variables (α in = 0.05 and α out = 0.10 with backward selection), and then we introduced GSH, SOD, nesfatin-1, UA and TBA to the new equation of Y = 2.437X_1_+0.045X_2_+0.025X_3_+0.062X_4_+1.658X_5_, R^2^ = 0.458 (Y: HOMA-β, X_1_: GSH, X_2_: SOD, X_3_: nesfatin-1, X_4_: UA, X_5_: TBA, *P* < 0.001), with the adjusted R^2^ = 0.442 (Supplementary Table 1), the results from this new equation showed that the standardized regression coefficients of GSH, SOD, nesfatin-1, UA and TBA were 0.300, 0.271, 0.284, 0.123, 0.112, respectively.

### Comparisons between subgroups of T2DM and prediabetes divided by HOMA-β

To further explore whether serum metabolic indexes could be affected by β-cell insulin secretion, we divided T2DM and prediabetes patients into subgroups by HOMA-β with the cut-off value of 62.9 for male and 60.6 for female (31). The characteristics of T2DM and prediabetes subgroups and the differential analyses were described in Table 3. The differences of age, gender and BMI for T2DM and prediabetes subgroups were non-significant. Further analysis revealed that serum levels of GSH, SOD and nesfatin-1 in T2DM or prediabetes with impaired HOMA-β values (under the cut-off) were apparently low (*P* < 0.001, *P* = 0.006, *P* < 0.001, respectively, in T2DM; *P* < 0.001, *P* < 0.001, *P* < 0.001, respectively, in prediabetes), compared to those patients with normal HOMA-β values (equal to or above the cut-off). Moreover, RBP and TIBC levels in T2DM subgroup with normal HOMA-β were obviously reduced (*P* = 0.05, *P* < 0.001, respectively), compared to those with impaired HOMA-β, but this reduction was not observed between the two prediabetes subgroups.

**Table 3:** Anthropometric and clinical characteristics of the subgroups divided by HOMA-β.

**Parameters**	**T2DM**		**|t|**	***P***	**Prediabetes**		**|t|**	***P***
	**HOMA-β reduced**	**HOMA-β normal**			**HOMA-β reduced**	**HOMA-β normal**		
Age (years)^a^	54 (51–61)	55 (51–61)	0.152	0.880	55 (51.50–57)	57 (51.75–62)	1.595	0.116*
Gender (M/F)	32/25	9/9	0.653	0.419*	10/19	33/15	0.015	0.903*
BMI (Kg/m^2^)^b^	23.16 ± 4.31	22.51 ± 2.02	0.617	0.539	22.97 ± 2.85	23.37 ± 2.66	0.585	0.561
GSH (μmol/L)^b^	7.54 ± 2.90	14.02 ± 5.42	4.854	<0.001	8.70 ± 3.02	14.58 ± 7.47	4.404	<0.001
SOD (U/ml)^b^	1,557.35 ± 181.60	1,695.31 ± 171.13	2.847	0.006	1,846.28 ± 257.13	2,075.24 ± 156.00	4.237	<0.001
Nesfatin-1 (pg/ml)^b^	564.55 ± 171.70	863.40 ± 299.85	4.025	<0.001	598.41 ± 227.24	1,087.64 ± 672.25	4.184	<0.001
ADPN (μg/ml)^b^	16.79 ± 12.20	23.06 ± 19.17	1.64	0.105	65.86 ± 11.85	63.46 ± 13.60	0.757	0.452
RBP (mg/L)^b^	87.74 ± 33.38	106.69 ± 40.59	1.991	0.05	33.18 ± 14.77	32.67 ± 19.67	0.116	0.908
TIBC (μmol/L)^b^	77.98 ± 67.46	33.48 ± 8.14	4.869	<0.001	38.49 ± 8.93	40.66 ± 7.94	1.046	0.299
NAGL (ng/ml)^b^	443.84 ± 230.19	471.46 ± 245.38	0.437	0.663	1,296.21 ± 347.49	1,381.25 ± 379.01	0.943	0.349
CysC (mg/L)^b^	0.78 ± 0.62	1.19 ± 1.05	1.577	0.130	0.68 ± 0.14	0.71 ± 0.17	0.949	0.346
HDL-C (mmol/L)^b^	1.13 ± 0.30	1.27 ± 0.24	1.801	0.076	1.41 ± 0.28	1.24 ± 0.24	2.552	0.013
LDL-C (mmol/L)^b^	3.36 ± 0.93	3.13 ± 1.17	0.832	0.488	3.79 ± 0.63	3.43 ± 0.78	2.038	0.046
HDL-C/LDL-C^b^	0.36 ± 0.13	0.49 ± 0.34	1.545	0.139	0.38 ± 0.09	0.38 ± 0.11	0.047	0.963
TBA (μmol/L)^b^	3.12 ± 2.38	4.62 ± 5.07	1.213	0.240	3.07 ± 3.26	3.88 ± 4.38	0.834	0.407
UA (μmol/L)^b^	342.12 ± 95.51	371.31 ± 161.53	0.728	0.475	328.98 ± 72.86	391.73 ± 88.37	3.102	0.003
Scr (μmol/L)^b^	1.17 ± 1.49	2.01 ± 2.51	1.352	0.191	0.87 ± 0.12	0.96 ± 0.19	2.562	0.013
BUN (mmol/L)^b^	8.34 ± 19.07	8.92 ± 7.15	0.125	0.901	5.40 ± 1.04	5.24 ± 1.21	0.584	0.561
HbA1c (%)^b^	7.93 ± 1.75	7.49 ± 2.42	0.850	0.850	5.89 ± 0.31	5.87 ± 0.33	0.237	0.813
FBG (mmol/L)^b^	9.00 ± 3.70	6.32 ± 0.99	4.949	<0.001	5.93 ± 0.36	5.80 ± 0.46	1.325	0.190
2h-BG (mmol/L)^b^	14.40 ± 4.64	11.45 ± 2.90	2.450	0.017	8.44 ± 1.47	8.82 ± 1.61	0.983	0.329
Insulin (μIU/ml)^b^	5.49 ± 2.29	11.22 ± 4.14	5.598	<0.001	5.24 ± 1.46	12.75 ± 6.89	6.520	<0.001
HOMA-IR^b^	2.12 ± 1.02	3.30 ± 1.77	2.665	0.015	1.39 ± 0.45	3.30 ± 1.77	6.386	<0.001
HOMA-β^b^	25.34 ± 15.09	80.68 ± 17.89	12.967	<0.001	43.23 ± 9.69	113.87 ± 68.58	6.268	<0.001
HOMA-IS^b^	63.00 ± 49.00	39.00 ± 21.00	2.082	0.041	0.79 ± 0.25	37.00 ± 16.00	8.188	<0.001

^a^Median (interquartile range), ^b^mean ± SD, ^*^Chi-Square P < 0.05. BMI, body mass index; GSH, glutathione; SOD, superoxide dismutase; ADPN, adiponectin; RBP, retinol binding protein; TIBC, total iron binding capacity; NAGL, neutrophil gelatinase-associated lipocalin; CysC, cystatin C; HDL-C, high density lipoprotein cholesterol; LDL-C, low density lipoprotein cholesterol; TBA, total bile acid; UA, uric acid; Scr, serum creatinine; BUN, blood urea nitrogen; HbA1c, glycosylated hemoglobin; FBG, fasting blood-glucose; 2h-PG, two-hour post glucose; HOMA-IR, homeostasis model assessment of insulin resistance; HOMA-β, homeostasis model assessment of β-cell; HOMA-IS, homeostasis model assessment of insulin sensitivity.

### Comparisons of HOMA-β among subgroups of IFG, IGT and IFG+IGT in prediabetes

To assess whether β-cell insulin secretion varies in prediabetes, three subgroups of IFG, IGT and IFG combined IGT were divided, according to the ADA classification, and their HOMA-β values were compared. The basic characteristics of the subgroups and the comparisons were summarized in the Supplementary Table 2. HOMA-β values in the subgroup of IGT seemed higher (113.52 ± 100.03) than that in IFG (75.00 ± 37.41) or IFG+IGT (74.08 ± 48.55), but the difference among the three subgroups was non-significant (*P* = 0.096). Serum levels of SOD in the IGT subgroup (2,090.95 ± 154.00 U/ml) were significantly higher than that in the IFG combined IGT subgroup (1,869.69 ± 330.96 U/ml), but no apparent difference was found when compared to the IFG subgroup (1,999.77 ± 195.08 U/ml). In contrast, levels of GSH (*P* = 0.502), nesfatin-1 (*P* = 0.793), ADPN (*P* = 0.724), TIBC (*P* = 0.263) and NAGL (*P* = 0.808) were insignificant among the subgroups of IGT, IFG and IFG+IGT.

## Discussion

To the best of our knowledge, this research is the first to reveal the differences of circulating levels of nesfatin-1, GSH and SOD in a progressive direction from the healthy condition to T2DM patients through prediabetes. We also disclosed the correlation between HOMA-β and the biomarkers of nesfatin-1, GSH and SOD, and found that these factors could exert influence on β-cell secretion.

Comprised of glutamate, cysteine and glycine, GSH is a ubiquitous thiol tripeptide which could consume hydroxyl, peroxynitrite and superoxide radicals through interacting with ROS (32). Glutathione peroxidases are prominent enzymes in protecting cells against oxidative stress by oxidizing GSH to glutathione and depleting the radicals (33). A previous study observed GSH deficiency in T2DM patients (34), while another report unfolded slightly higher GSH levels in IFG than in the control (35). In the present research, we found that serum GSH levels in T2DM were significantly reduced than that in prediabetes or the control, and this significant reduction was also confirmed in prediabetes vs. the control; further comparisons revealed that the difference of GSH levels among prediabetes subgroups of IGT, IFG and IFG+IGT was insignificant (*P* = 0.502). Notably, GSH levels in either subgroup of T2DM or prediabetes with impaired HOMA-β values were overwhelmingly dropped, in contrast to the counterparts with normal HOMA-β. As a substrate of glutathione peroxidase, GSH is of great importance in human metabolic activities for it constitutes the anti-oxidative defensive system *in vivo*. SOD is an antioxidant enzyme, capable of catalyzing superoxide to hydrogen peroxide and oxygen molecules (36). There are three isoforms of mammal SOD: SOD1 in cytosolic (such as Cu and Zn-SOD), SOD2 in mitochondrion (such as Mn-SOD) and SOD3 in extracellular matrix (such as EC-SOD) (37). In this study, the SOD detected in serum mainly belongs to SOD3. Our results demonstrated that SOD levels in T2DM and prediabetes were remarkably decreased compared with the healthy control, and we also observed a significant reduction of the SOD level in T2DM vs. prediabetes. GSH and SOD are the classical component of the cell anti-oxidation system. Indeed, our results revealed that GSH and SOD levels in the subgroup of T2DM or prediabetes with impaired β-cell insulin secretion were significantly low in comparison to the counterpart with normal insulin action; in addition, serum SOD levels in subgroup of IFG or IFG combined IGT displayed a marked reduction compared to the IGT subgroup. Our results of GSH and SOD reduction in T2DM and prediabetes suggest that in the condition of T2DM or prediabetes, the anti-oxidation capacity in the body may be partly damaged, which was in consistent with previous studies (2, 38, 39).

Elevated blood glucose is essential for the formation of advanced glycation end products (AGEs), a group of modified proteins and/or lipids with damage potential, which contribute to the progression of T2DM. For one thing, AGEs could increase the formation of ROS and undermine the anti-oxidative defense mechanism of human body; for another, the generation of AGEs is enhanced under oxidative stress conditions (39). Abnormal glycometabolism is the major hallmark for the pathogenesis and development of T2DM, which is currently controllable but irreversible in most cases. However, prediabetes is a reversible state that could be transited from disturbance of carbohydrate metabolism to normoglycaemia; therefore, fortifying the antioxidative defense system of the patients with prediabetes may help regress or alleviate the progression of the disease toward T2DM.

Compared to IFG, IGT presents severe transitory hyperglycemia, which may explain the higher GSH, SOD, nesfatin-1, insulin and HOMA-β levels in IGT than IFG in our study. Investigation on nesfatin-1 provided evidences that its circulating level correlated with T2DM and elevated in newly diagnostic T2DM patients (24), but decreased in those patients who received antidiabetic treatment (28). In our study, serum nesfatin-1 levels in T2DM were obviously reduced compared to that in prediabetes or healthy subjects, which was supported by other evidences (40, 41), and this reduction still presented when comparing prediabetes to the control, which has not been reported so far. Furthermore, we found that difference of serum nesftain-1 levels in IGT were insignificant compared to either in IFG or in IFG+IGT. A few studies (20, 42, 43) declared the antioxidant function of nesfatin-1; noteworthy, we observed that nesfatin-1levels were significantly correlated with GSH (*r* = 0.222, *P* = 0.003) and SOD (*r* = 0.287, *P* < 0.001) (refer to the Supplementary Figure), indicating a high probability of nesfatin-1 exerting antioxidative effects in the development of T2DM.

Our exploration of impact factors on β-cell secretory action revealed that FBG, insulin and HOMA-IR could significantly affect insulin secretion, as well as the factors of GSH, nesfatin-1 and SOD. β-cell viability and insulin release could be crippled as a consequence of hyperglycaemia and glucotoxicity in human body (44, 45). Oxidative stress has been widely accepted as a major causative factor responsible to increase the production of ROS and impede the antioxidant pathway combined with glucotoxicity and/or lipotoxicity, ultimately leading to β-cell dysfunction and overt T2DM (45, 46). GSH and SOD were recognized as vital components of intrinsic defense mechanism involving anti-oxidative activity (10), and higher circulating GSH and SOD levels were believed to be able to protect β-cells from damage of free radicals, including ROS, superoxide, hydrogen peroxide and hydroxyl (6).

To make a long story short, our study successfully managed to identify the correlation of nesfatin-1, GSH and SOD levels with β cell dysfunction in T2DM, implicating their roles in β cell toxicity as a result of oxidative stress. However, this study is limited firstly in that it is cross-sectional in nature and unable to determine causality between the disease and its risky factors in diabetic patients. Secondly, due to our relatively small sample size of only 75 T2DM and 67 prediabetes individuals, further investigation with enlarged samples is needed to make the conclusion more convincible. Thirdly, the cutoffs used for HOMA-β evaluation were adopted from a previous study based on population in Tehran, and the impact of the ethnic variations on glycemic indices has to be considered when apply the data to different population. Fourthly, medication histories of the studied subjects were not obtained because of the unavailability of sufficient clinical information of the patients at hand. To remedy this, we plan to record the medication history details in our future cohort study on exploring whether insulin or other anti-diabetic agents can impose effects on nesfatin-1 level in serum. Last but not least, despite the fact that we revealed that serum nesfatin-1, GSH and SOD levels correlated with and affected insulin secretion, more efforts should be made to unveil the effects of these factors on insulin function.

## Data availability statement

The raw data supporting the conclusions of this article will be made available by the authors, without undue reservation.

## Ethics statement

The studies involving human participants were reviewed and approved by Ethics Committee of Xiangya Hospital of Central South University. The patients/participants provided their written informed consent to participate in this study.

## Author contributions

KH collected the data and wrote the main manuscript draft. YL and KW made the statistical analysis and the figures. JW and HL prepared the tables. BY designed the investigation, modified the original draft, and approved the final version. All authors reviewed the manuscript and agreed on this submission.

## Conflict of interest

The authors declare that the research was conducted in the absence of any commercial or financial relationships that could be construed as a potential conflict of interest.

## Publisher's note

All claims expressed in this article are solely those of the authors and do not necessarily represent those of their affiliated organizations, or those of the publisher, the editors and the reviewers. Any product that may be evaluated in this article, or claim that may be made by its manufacturer, is not guaranteed or endorsed by the publisher.

## Supplementary Material

The Supplementary Material for this article can be found online at: https://www.frontiersin.org/articles/10.3389/fpubh.2022.882686/full#supplementary-material
