# Supplementary material for: Influence of circulating nesfatin-1, GSH and SOD on insulin secretion in the development of T2DM
Source: Front Public Health. 2022 Aug 15;10:882686. doi: 10.3389/fpubh.2022.882686 (PMC9421132; doi:10.3389/fpubh.2022.882686)
Supplement: Supplementary file 1 [file Data_Sheet_1.docx]

**Supplementary Table 1** independent variables introduced to multiple liner regression except glycometabolism indexes

| Paraments | b | S_b_ | b’ | \|t\| | *P* |
| --- | --- | --- | --- | --- | --- |
| Constant | -94.869 | 19.664 | / | 4.825 | <0.001 |
| GSH | 2.437 | 0.496 | 0.300 | 4.918 | <0.001 |
| SOD | 0.045 | 0.010 | 0.271 | 4.402 | <0.001 |
| Nesfatin-1 | 0.025 | 0.005 | 0.284 | 4.716 | <0.001 |
| UA | 0.062 | 0.029 | 0.123 | 2.120 | 0.035 |
| TBA | 1.658 | 0.839 | 0.112 | 1.977 | 0.050 |

GSH: glutathione; SOD: superoxide dismutase; UA: uric acid; TBA: total bile acid.

**Supplementary Table 2** Anthropometric and clinical characteristics of IFG, IGT and IFG combined IGT subgroups

| Paraments | IFG | IGT | IFG+IGT | F | *P* |
| --- | --- | --- | --- | --- | --- |
| Age (years)^a^ | 55(52-59) | 54.5(50.25-63.50) | 56(51.25-58.75) | 0.064 | 0.938 |
| Gender(M/F) | 8/15 | 13/3 | 12/16 | 4.297 | 0.01* |
| BMI(Kg/m^2^) | 23.18±2.98 | 22.70±2.94 | 23.37±2.52 | 0.290 | 0.750 |
| GSH (µmol/L) | 11.97±7.18 | 15.05±16.15 | 11.66±5.82 | 0.697 | 0.502 |
| SOD(U/ml) | 1999.77±195.08 | 2090.95±154.00 | 1869.69±330.96 | 4.130 | 0.021 |
| Nesfatin-1(pg/ml) | 866.36±603.80 | 1023.46±648.50 | 906.52±839.22 | 0.233 | 0.793 |
| ADPN(µg/ml) | 63.20±14.06 | 66.59±7.60 | 64.37±14.34 | 0.324 | 0.724 |
| RBP (mg/L) | 41.23±31.45 | 28.58±11.35 | 39.22±28.48 | 1.176 | 0.315 |
| TIBC (µmol/L) | 40.13±31.45 | 61.02±55.12 | 48.64±43.15 | 1.362 | 0.263 |
| NAGL (ng/ml) | 1304.00±387.20 | 1372.74±354.93 | 1361.48±363.54 | 0.214 | 0.808 |
| CysC (mg/L) | 0.68±0.15 | 0.70±0.16 | 0.71±0.17 | 0.187 | 0.830 |
| TP (g/L) | 75.42±3.40 | 73.68±4.00 | 74.79±2.72 | 1.317 | 0.275 |
| ALB (g/L) | 46.36±2.54 | 45.58±2.63 | 45.43±1.70 | 1.156 | 0.321 |
| TG (µmol/L) | 2.16±1.54 | 1.86±1.36 | 1.97±0.93 | 0.290 | 0.749 |
| TC (µmol/L) | 5.73±0.81 | 4.75±0.79 | 5.63±0.96 | 7.023 | 0.002 |
| HDL-C (mmol/L) | 1.35±0.30 | 1.23±0.23 | 1.34±0.27 | 1.077 | 0.347 |
| LDL-C (mmol/L) | 3.78±0.65 | 3.08±0.62 | 3.71±0.75 | 5.621 | 0.006 |
| HDL-C/LDL-C | 0.38±0.08 | 0.42±0.11 | 0.37±0.12 | 1.314 | 0.276 |
| TB (µmol/L) | 14.17±6.01 | 13.77±4.75 | 14.10±5.09 | 0.029 | 0.971 |
| DB (µmol/L) | 3.70±1.54 | 3.99±1.60 | 1.09±0.21 | 0.206 | 0.814 |
| ALT (U/L) | 28.01±16.58 | 29.06±14.20 | 34.94±37.03 | 0.484 | 0.618 |
| AST (U/L) | 28.11±11.01 | 28.02±8.34 | 29.70±19.56 | 0.098 | 0.906 |
| TBA (µmol/L) | 3.53±5.20 | 4.83±5.22 | 3.15±3.47 | 0.710 | 0.496 |
| UA (µmol/L) | 328.52±88.49 | 432.18±68.23 | 369.83±102.86 | 6.150 | 0.004 |
| Scr (µmol/L) | 0.91±0.15 | 0.96±0.17 | 0.91±0.18 | 0.476 | 0.623 |
| eGFR (ml/min**^.^**1.73m^2^) | 77.91±11.24 | 85.78±13.09 | 79.29±12.14 | 2.196 | 0.120 |
| BUN (mmol/L) | 5.22±1.26 | 5.31±1.06 | 5.38±1.10 | 0.114 | 0.892 |
| HbA1c (%) | 5.76±0.26 | 5.82±0.40 | 6.01±0.27 | 4.769 | 0.012 |
| FBG (mmol/L) | 5.97±0.29 | 5.28±0.33 | 6.07±0.25 | 43.859 | 0.000 |
| 2h-BG (mmol/L) | 7.14±0.52 | 9.26±0.95 | 9.56±1.45 | 34.441 | 0.000 |
| Insulin (µIU/ml) | 9.08±4.17 | 10.02±9.20 | 9.54±6.34 | 0.099 | 0.906 |
| HOMA-IR | 2.40±1.08 | 2.37±2.20 | 2.58±1.73 | 0.114 | 0.893 |
| HOMA-β | 75.00±37.41 | 113.52±100.03 | 74.08±48.55 | 2.429 | 0.096 |
| HOMA-IS | 50.00±20.00 | 63.00±0.31 | 56.00±33.00 | 0.985 | 0.379 |

^a^ media (interquartile range), ^*^Chi-Square *P*<0.05. BMI: body mass index; GSH: glutathione; SOD: superoxide dismutase; ADPN: adiponectin; RBP: retinol binding protein; TIBC: total iron binding capacity; NAGL: neutrophil gelatinase-associated lipocalin; CysC: cystatin C; TP: total protein; ALB: albumin; TG: total triglycerides; TC: total cholesterol; HDL-C: high density lipoprotein cholesterol; LDL-C: low density lipoprotein cholesterol; TB: total bilirubin; DB: direct bilirubin; ALT: alanine aminotransferase; AST: aspartate aminotransferase; TBA: total bile acid; UA: uric acid; Scr: serum creatinine; eGFR: estimated glomerular filtration rate; BUN: blood urea nitrogen; HbA1c: glycosylated hemoglobin; FBG: fasting blood-glucose; 2h-PG: two-hour post glucose; HOMA-IR: homeostasis model assessment of insulin resistance; HOMA-β: homeostasis model assessment of β cell; HOMA-IS: homeostasis model assessment of insulin sensitivity; Ucr: urine creatinine; UmALB: urine microalbumin; uACR: urinary microalbumin creatinine ratio.
