# Supplementary figures and images for: Influence of circulating nesfatin-1, GSH and SOD on insulin secretion in the development of T2DM
Source: Front Public Health. 2022 Aug 15;10:882686. doi: 10.3389/fpubh.2022.882686 (PMC9421132; doi:10.3389/fpubh.2022.882686)

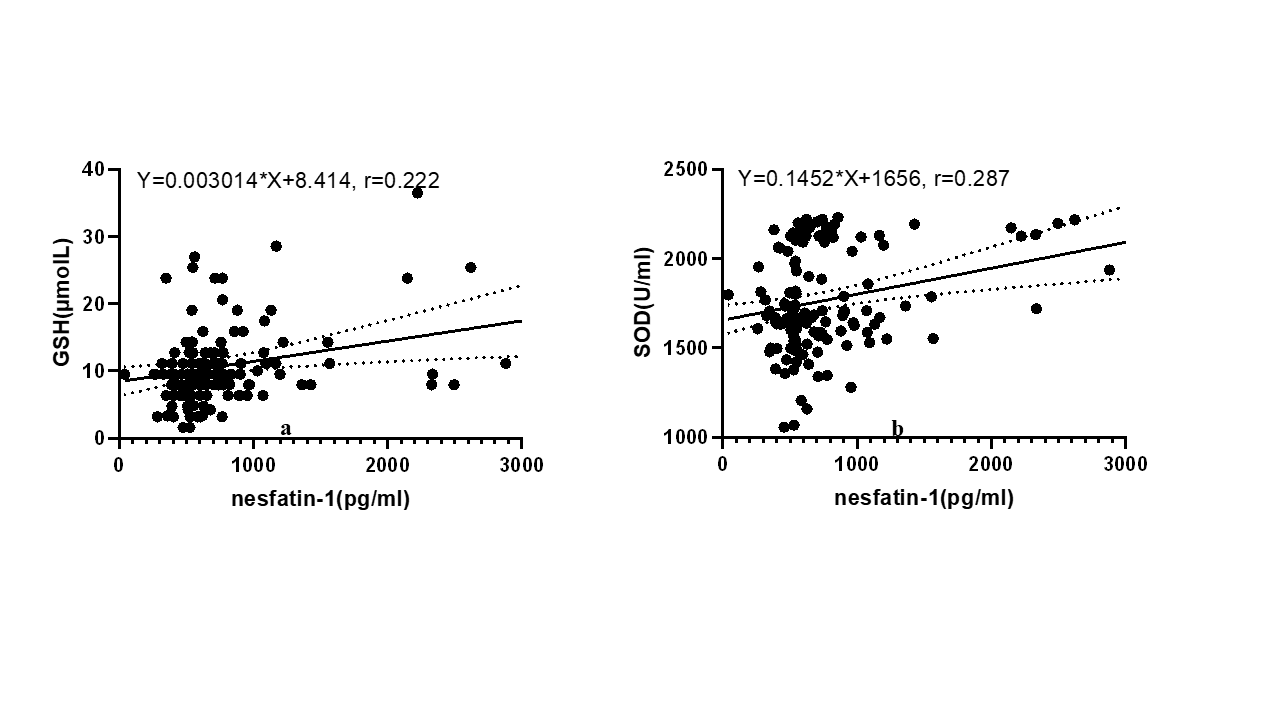

Supplement: Supplementary file 2 [file Image_1.TIF]
